# Chromosome-level genome assembly and population genomics unveil strigolactone-regulated growth adaptation in the mycoheterotrophic orchid *Gastrodia elata*

**DOI:** 10.1093/hr/uhag099

**Published:** 2026-06-04

**Authors:** Zhongyi Hua, Lihong Li, Yuchao Chen, Yiying Cao, Wei Liu, Xiying Teng, Junhui Zhou, Yuyang Zhao, Yuan Yuan

**Affiliations:** State Key Laboratory for Quality Ensurance and Sustainable Use of Dao-di Herbs, Experimental Research Center, China Academy of Chinese Medical Sciences, Beijing 100700, China; School of Pharmacy, Jiangsu University, Zhenjiang 212013, China; State Key Laboratory for Quality Ensurance and Sustainable Use of Dao-di Herbs, Experimental Research Center, China Academy of Chinese Medical Sciences, Beijing 100700, China; Agricultural Biotechnology Center, Ningxia Academy of Agriculture and Forestry Sciences, Yinchuan 750002, China; National Resource Center for Chinese Materia Medica, China Academy of Chinese Medical Sciences, Beijing 100700, China; State Key Laboratory for Quality Ensurance and Sustainable Use of Dao-di Herbs, Experimental Research Center, China Academy of Chinese Medical Sciences, Beijing 100700, China; State Key Laboratory for Quality Ensurance and Sustainable Use of Dao-di Herbs, Experimental Research Center, China Academy of Chinese Medical Sciences, Beijing 100700, China; National Resource Center for Chinese Materia Medica, China Academy of Chinese Medical Sciences, Beijing 100700, China; National Resource Center for Chinese Materia Medica, China Academy of Chinese Medical Sciences, Beijing 100700, China; State Key Laboratory for Quality Ensurance and Sustainable Use of Dao-di Herbs, Experimental Research Center, China Academy of Chinese Medical Sciences, Beijing 100700, China

## Abstract

Mycoheterotrophic plants rely entirely on fungal symbionts for nutrients, yet the role of intraspecific genomic variation in shaping symbiotic adaptation remains unclear. *Gastrodia elata* is a mycoheterotrophic orchid with multiple cultivated varieties. Here, we generated a chromosome-level genome of *G. elata* Bl. f. *glauca.* Comparative genomic analyses with published *G. elata* assemblies revealed extensive intraspecific variation, characterized by transposon-mediated inversions occurring in 26% of syntenic regions. Notably, these regions frequently harbored orphan genes. Population genomic analysis of 150 individuals identified three genetically distinct clades: two cultivated (Clades E and G) and one hybrid (Clade I). Transcriptomic profiling uncovered clade-specific expression patterns in symbiosis-related genes, particularly within strigolactone signaling pathways. Molecular dynamics simulations and protein interaction assays demonstrated that polymorphisms in the M domain of the suppressor protein DWARF53 (GeD53) modulate strigolactone signaling by altering the stability of its interaction with the receptor (GeD14). Specifically, a Clade G-specific haplotype enhanced signaling through stabilized protein interactions, thereby influencing tuber development genes, whereas GeD14 variants had minimal functional impact. Further co-expression networks identified *LOL5*, *RNP1*, and *MTHD* as downstream effectors correlating with clade-specific tuber phenotypes and carbohydrate allocation. These findings demonstrate how intraspecific variation in strigolactone signaling components drives functional divergence in *G. elata*, providing both mechanistic insights into mycoheterotrophic adaptation and genomic resources for future research.

## Introduction

Most plants are autotrophic, synthesizing carbohydrates from carbon dioxide via photosynthesis. However, a subset of plants has evolved heterotrophic lifestyles, acquiring nutrients from other organisms. These heterotrophic plants have long fascinated biologists due to their unique evolutionary adaptations [1, 2], signaling mechanisms [3, 4], and roles in nutrient cycling [5, 6].

Genomic studies of heterotrophic plants have uncovered both convergent and divergent evolutionary trajectories. For instance, parasitic plant genomes, such as *Cuscuta campestris* [7] and *Cuscuta australis* [8], exhibit significant gene loss, especially in pathways essential for photosynthesis and soil-based nutrient absorption, reflecting their reliance on host plants. The *C. campestris* genome has also provided the first evidence of horizontal gene transfer from the host to the parasite, demonstrating cross-species genetic exchange [7]. Similarly, the genome of *Sapria himalayana*, whose flowers emerge directly from its host, revealed that the degree of gene loss correlates with the extent of parasitism [9].

Even more striking are the genomes of fully non-photosynthetic mycoheterotrophic plants (MHPs), which combine the complete loss of photosynthetic genes with intricate mechanisms for fungal symbiosis. *Gastrodia elata*, a mycoheterotrophic orchid dependent on *Armillaria* fungi, exemplifies this strategy. Its genome retains and expands key functional modules, including glycoside hydrolases for fungal cell wall degradation and strigolactone (SL) signaling components for symbiotic interaction, despite losing much of the photosynthetic machinery [10]. These patterns are reinforced by recent studies. For example, genomic data from *Platanthera* orchids suggest that MHPs may use fungal-derived trehalose as a primary carbon source, supported by the widespread expansion of the trehalase gene across MHP lineages [11]. Regarding signaling, SLs, originally identified as facilitators of arbuscular mycorrhizal (AM) symbiosis [12], have also been implicated in *G. elata’s* regulation of *A. gallica* growth via reactive oxygen species [13]. However, whether these symbiotic signaling pathways exhibit intraspecific variation that contributes to the diverse growth strategies among *G. elata* cultivars remains largely unexplored.

Although several chromosome-level assemblies of *G. elata* have been generated in recent years [14–16], these resources lack clear varietal specification. In *G. elata*, there are five major cultivated varieties characterized by distinct symbiotic and growth traits [17]. While population genomics has been instrumental in deciphering the genetic basis of intraspecific adaptation in autotrophic plants [18], its application in heterotrophic systems remains limited [19, 20]. Moreover, the absence of variety-specific reference genomes, coupled with a lack of integration among existing genomic datasets, complicates the precise characterization of genomic features linked to the divergent symbiotic adaptations observed across different clades.

To address these gaps, we generated a chromosome-level genome assembly for *G. elata* Bl. f. *glauca* to serve as a high-quality variety-specific reference. By integrating this assembly with population-scale resequencing and transcriptomic data, we aimed to delineate the genetic architecture of cultivated *G. elata* and characterize the genomic variations underlying divergent symbiotic traits. Advancing beyond previous species-level descriptions, this study resolves transposon-mediated structural variations (SVs) and how functional polymorphisms in the SL signaling GeD14-GeD53 module contribute to phenotypic variation across different *G. elata* clades. These findings provide a framework for exploring the evolutionary mechanisms and physiological trade-offs that characterize mycoheterotrophic adaptation.

## Results

### Genome sequencing, assembly, and annotation of *G. elata* Bl. f. *glauca*

To assemble the genome of *G. elata*, a total of 236.96 Gb (∼220.45× coverage) of PacBio SMRT reads, 170.67 Gb (∼103.41×) of Illumina short reads, and 139.34 Gb (∼129.62×) of Hi-C reads were generated. Initial assembly using Falcon resulted in a total contig size of 1.07 Gb across 973 contigs, with a contig N50 of 5.86 Mb and a maximum contig length of 24.63 Mb (Table 1). Subsequent scaffolding with Hi-C data via the 3D-DNA pipeline produced a chromosome-level assembly with a final total length of 1.05 Gb. This assembly comprised 18 pseudochromosomes, ranging from 35.84 to 132.89 Mb, with a scaffold N50 of 54.41 Mb and an anchoring rate of 98.49% (Fig. S1).

**Table 1:** Comparative statistics of *G. elata* genome assemblies.

	**Yuan *et al.* [10]**	**Chen *et al*. (2020)** **[21]** ^**a**^	**Xu *et al*. [16]**	**Bae *et al.* [15]^a^**	**Wang et al. (2023)[22]**	**This work**
Level	Scaffold	Scaffold	Chromosome	Chromosome	Chromosome	Chromosome
Assembled genome (Gb)	1.06	1.11	1.04	1.02	1.04	1.07
Anchored genome (Gb)			1.03	1.01	1.02	1.05
Anchored percent (%)			99.09	96.40	98.65	98.49
Contig N50	68.9 Kb	110 Kb		9.18 Mb	16.8 Mb	5.86 Mb
Scaffold N50 (Mb)	4.9	1.64	21.33	50.6	51.20	54.41
Repeat region proportion (%)	66.18	69.81	66.36	74.92	77.94	69.41
Number of *N*	35 512 911		78 011	14 200	90 000	68 600
Gene number	18 950	24 484	21 115	18 844	17 895	16 979
Number of BUSCO	1614	248	1614	1440	1614	1614
Complete BUSCO^b^	1198	303	1224	935	1226	1222
Percentage of complete BUSCO^b^	74.30	81.85	75.9	64.90	75.90	75.70

*a.* Raw sequencing data were not publicly available; therefore, the data reported in the original study were used directly.
*b.* ``Complete BUSCOs'' comprise both single-copy and duplicated complete BUSCO genes.

Repetitive elements were characterized, showing that interspersed repeats occupy 69.41% (743.47 Mb) of the *G. elata* genome (Table S1). Retrotransposons were the dominant class, with long terminal repeat (LTR) retrotransposons accounting for 49.71% of the total assembly, primarily represented by the *Gypsy* (38.07%) and *Copia* (1.41%) families. DNA transposons comprised 11.36% of the genome, where those characterized by terminal inverted repeats (TIRs), such as *Mutator* (6.35%) and *CACTA* (3.27%), were the most prevalent. Other components included Helitrons (3.73%) and non-LTR retrotransposons (0.06%), as detailed in Table S1.

**Figure 1:**
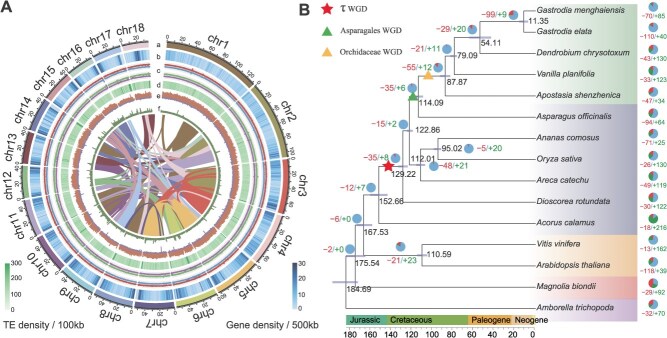
Overview of the chromosome-level genome of *G. elata* Bl. f. *glauca*. (A) Circos map showing the genomic landscape of *G. elata* Bl. f. *glauca* genome. From outermost to innermost ring: a—chromosome length; b—gene density; c—the ratio of nucleotides, with red representing A, blue representing C, green representing T, and purple representing G; d—TE density; e—Class I elements (retrotransposons), specifically LTR density, with red representing *Gypsy* and purple representing *Copia*; f—Class II elements (DNA transposons), specifically TIR density. (B) Divergence time estimation and gene family expansion/contraction analyses. The τ WGD event, the Orchidaceae WGD, and the Asparagales WGD are indicated by a red star, yellow triangle, and green triangle, respectively. Pie charts show the proportions of expanded (red), contracted (blue), and unchanged (green) gene families; adjacent numbers indicate the counts of expanded/contracted families.

Gene annotation was performed using a combined approach incorporating *ab initio* prediction, protein homology, and transcriptome evidence, resulting in the identification of 22 931 structural genes (Fig. 1A). Among these, 5952 were classified as pseudogenes based on protein homology and lack of expression (Fig. S2), yielding a final set of 16 979 protein-coding genes (Table 1). Of these, 16 876 genes (99.39%) were successfully anchored to chromosomes, and 91.41% were functionally annotated based on InterPro, Swiss-Prot, KEGG, and Gene Ontology (GO) databases (Table S2).

To assess assembly quality, Illumina paired-end reads were mapped back to the genome, achieving a mapping rate of 98.79% (Table S3). Benchmarking Universal Single-Copy Orthologs (BUSCO) analysis using the embryophyta_odb10 dataset showed that 75.70% of BUSCO were complete, 2.4% were fragmented, and 21.9% were missing (Table S4). This missing rate is consistent with other mycoheterotrophic species and likely reflects the evolutionary loss of conserved genes associated with the transition to a non-photosynthetic lifestyle, rather than incomplete assembly. These results collectively indicate a high-quality assembly.

Comparative genomics analysis across 15 plant species revealed that monocots and dicots diverged ~167.53 million year ago (Mya) (95% CI: 159.14–174.16 Mya), consistent with previous estimates. Within the *Gastrodia* genus, divergence between *G. elata* and *G. menghaiensis* was estimated at 11.35 Mya (95% CI: 7.09–16.01 Mya) (Fig. 1B). To investigate the whole-genome duplication (WGD) history of *G. elata*, 741 paralogous gene pairs (Fig. S3A) were identified. After filtering out tandem duplicates, the synonymous substitution rates (*K_s_*) of 434 paralogous gene pairs were calculated and compared with those of related species. The absence of a discernible *K_s_* peak in *Acorus calamus* corroborated prior findings that this species has not experienced any WGD event [23]. A shared *K_s_* peak between 1.0 and 1.5 was observed in *G. elata* and *Dendrobium chrysotoxum* (Fig. S3B). Notably, this peak value was smaller than the *K_s_* peak corresponding to the divergence of *G. elata* and *Asparagus officinalis*, yet larger than that corresponding to the divergence of *G. elata* and *D. chrysotoxum*, indicating that it represents a common Orchidaceae-specific WGD event [24]. In contrast, neither the τ WGD event [23] nor the Asparagales-common WGD event [25] left detectable signatures in the *G. elata* genome.

The common ancestor of the *Gastrodia* genus experienced widespread gene family contraction, with 99 families contracted and only nine expanded. *G. elata* alone exhibited contraction of 110 gene families. KEGG enrichment of expanded families revealed involvement in carbohydrate metabolism, cytochrome P450 activity, and glutathione metabolism (Fig. S4A). Further expansions during *Gastrodia*’s diversification were enriched in phenylpropanoid biosynthesis and plant hormone signaling pathways (Fig. S4B). In contrast, contracted families were enriched for transcription factors such as ERF087 and TCP11, leucine-rich repeat receptors, and disease resistance, genes including *LRK10* and *PR1C* (Tables S5 and S6).

### Transposon-induced inversions contribute to *G. elata* intraspecies variation

**Figure 2:**
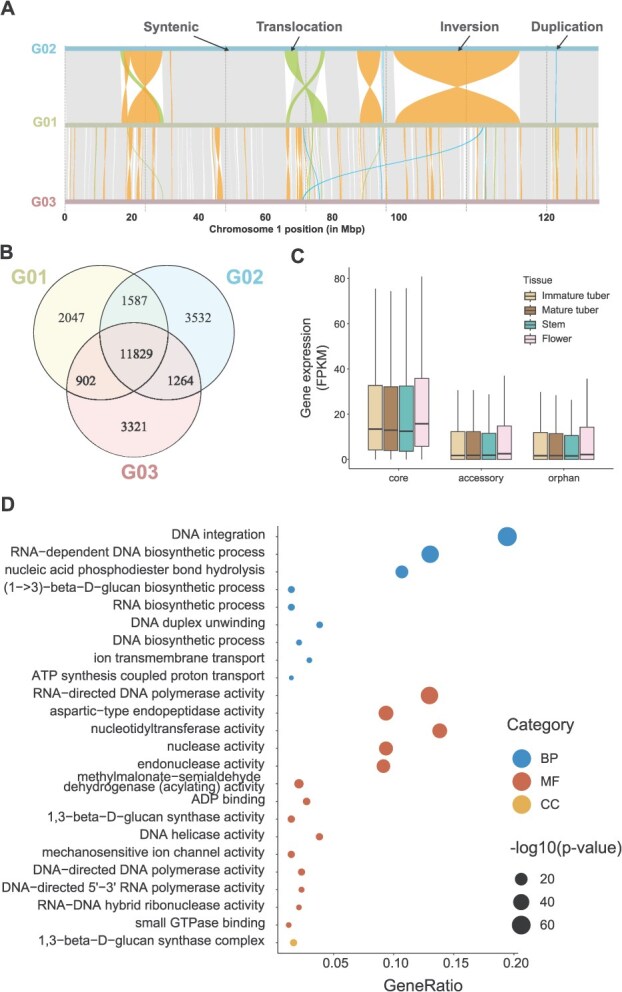
Comparative genomics of *G. elata* Bl. f. *glauca*. (A) SVs observed on Chromosome 1 across the *G. elata* intraspecific pan-genome. (B) Venn diagram showing shared orthologous gene families within the pan-genome. (C) Expression levels of core, accessory, and orphan genes across the pan-genome. (D) The GO enrichment analysis of orphan genes.

To investigate intraspecific SVs, the newly assembled *G. elata* genome (G01) was compared with two publicly available assemblies: G02 [16] and G03 [14] (Table 1). Between G01 and G02, 674.52 Mb of syntenic regions and 330.00 Mb of rearranged regions were detected, including 264.78 Mb of inversions, 31.11 Mb of translocations, and 34.10 Mb of translocated inversions (Fig. S5, Table S7). Comparison with G03 revealed 849.34 Mb of syntenic regions and 53.67 Mb of inversions (Fig. S5, Table S8). The largest SV was a 31-Mb inversion on Chr1 (82–113 Mb) between G01 and G02 (Fig. 2A), supported by Hi-C interaction maps that ruled out assembly artifacts (Fig. S6). Analysis of the flanking regions identified a significantly higher transposable element (TE) density at inversion breakpoints (mean = 78.21%) compared to the genomic background (mean = 67.40%; *P* < 0.001, Wilcoxon rank-sum test; Fig. S7), indicating that these rearrangements are predominantly transposon-mediated. In addition to inversions, other SVs, including presence/absence variations (PAVs) and copy number variations (CNVs), were also systematically identified across three *G. elata* assemblies. A total of 2.61 Mb of presence and 3.50 Mb of absence sequences were identified between G01 and G02, along with 19.48 Mb of segmental gains and 9.05 Mb of segmental losses (Table S7). Similarly, the comparison between G01 and G03 revealed 9.66 Mb of sequence presence and 8.74 Mb of sequence absence, accompanied by 1.24 Mb of segmental gains and 3.04 Mb of segmental losses (Table S8). Notably, while these individual sequence-level fluctuations are substantial, they largely offset each other, resulting in a highly dynamic genomic landscape with minimal net changes in overall genome size.

To further explore the evolutionary impact of these SVs, we examined the distribution of gene families. Gene families shared across all three assemblies were defined as core genes, whereas those unique to a single assembly were designated as orphan genes (Fig. 2B). Core genes represented 69.67% of annotated genes in G01 (Table S9). Of the 16 482 G01 genes located in syntenic regions, 13 007 resided in syntenic regions and 3219 in inversion regions. Consequently, the gene count ratio between the syntenic and inverted regions was 4.04, compared to a region length ratio of 2.04. Core genes were significantly enriched in syntenic regions, while orphan genes were overrepresented in inversion regions (Table S10). Due to the relatively short length of most identified PAVs and CNVs (typically ranging from several hundred to 1000 bp), only genes whose coding sequences (CDS) were directly intersected by these variants were investigated. A total of 276 and 83 genes were identified as being affected by PAVs and CNVs, respectively (Table S11). Functional profiling of these specific gene sets through GO and KEGG annotation yielded no statistically significant enrichment results.

Expression profiling across tissues showed that core genes had higher expression levels than accessory and orphan genes (Fig. 2C, Table S12). GO enrichment analysis revealed that orphan genes are predominantly involved in DNA integration and RNA-directed DNA polymerase activity (Fig. 2D), processes associated with TEs. Notably, orphan genes such as *Gel01G00293*, *Gel01G00384*, and *Gel01G00606* encoded Ty3-I Gag-Pol polyproteins (Tables S9 and S13), implicating transposons as key drivers of genome variation.

### Population genomic structure and genetic diversity in cultivated *G. elata*

Whole-genome resequencing of 150 *G. elata* individuals yielded a total of 854.92 Gb of high-quality clean data, with an average sequencing depth of 5.33× per individual (Table S14), generating 14.3 million high-quality single nucleotide polymorphisms (SNPs), with an average density of 13.34 SNPs per Kb. Among these, 39.6% were located in protein-coding regions (Table S15). Kinship analysis revealed that 99.4% of sample pairs were genetically unrelated. Two sample pairs (H32-H30 and H40-H30) showed first-degree relatedness (kinship coefficient > 0.177) and were excluded from downstream analyses (Fig. S8).

**Figure 3:**
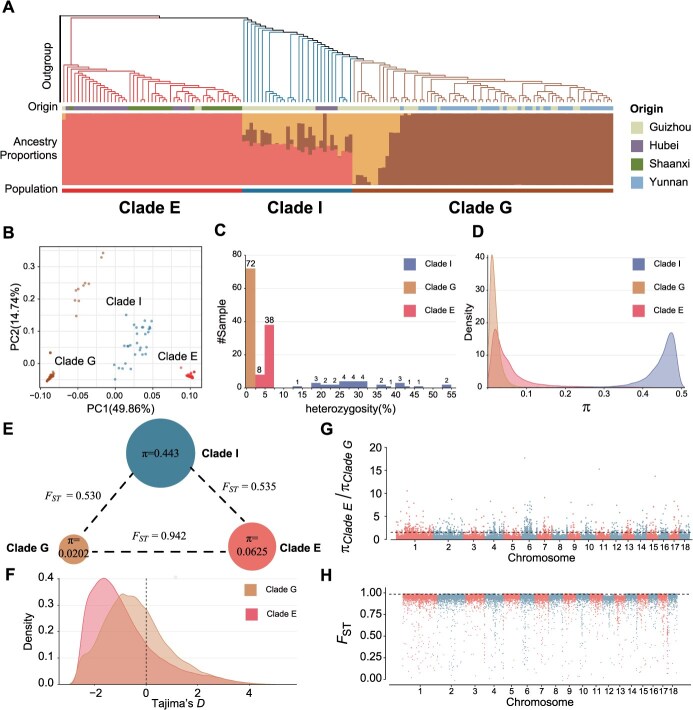
Population genomic structure of 150 *G. elata* individuals. (A) Maximum-likelihood phylogenetic tree. From top to bottom: geographic origin, population structure with cluster number *K* = 3, and population classified. (B) PCA of the *G. elata* populations. (C) Distribution of individual heterozygosity across clades. (D) Density distribution of nucleotide diversity (π) across clades. (E) Comparison of nucleotide diversity (π) and population differentiation (*F*_ST_) across three clades. Values within each circle indicate nucleotide diversity, and values along lines indicate *F*_ST_ between clades. (F) Distribution of Tajima’s *D* values for Clade E and Clade G. (G) Selective sweep signals inferred from the π ratio (π_Clade E_/π_Clade G_). (H) Selective sweep signals inferred from *F*_ST_ between Clade E and Clade G.

Population structure analyses resolved three distinct clades based on a hierarchical classification framework. The maximum-likelihood phylogenetic tree initially partitioned the 150 individuals into two primary lineages (Fig. 3A). By integrating this framework with ADMIXTURE analysis (Fig. S9), we defined the first lineage as Clade E, which was characterized by high genomic purity with >95% ancestry from a single genetic component (Fig. 3A). Within the second lineage, individuals entirely lacking the E-type component were categorized as Clade G. The remaining individuals in this lineage, which possessed ~50%–60% E-type ancestry and thus fell below the 90% threshold, were identified as a hybrid lineage, Clade I (Fig. 3A). This classification was robustly supported by principal component analysis (PCA), which explained 49.86% (PC1) and 14.74% (PC2) of the total genetic variation and clearly separated the samples into three distinct spatial clusters (Fig. 3B). Detailed information regarding the clade assignment of each sample is provided in Table S16.

Genetic diversity varied markedly among these clades, further validating their genomic status. Clades G and E exhibited low heterozygosity, with peaks at 2.4% and 6.1%, respectively, and minimal nucleotide diversity (π < 0.1 in 10-kb windows). In contrast, Clade I showed elevated heterozygosity (>10%) and much higher π values with a peak near 0.45 (Fig. 3C and D). The average π values were 0.0202 (Clade I), 0.0625 (Clade E), and 0.0530 (Clade G). Pairwise genetic distances between Clade I and Clades E or G were 0.535 and 0.530, respectively, while the highest differentiation was observed between Clade E and Clade G (Fig. 3E). Negative Tajima’s D values in Clades E and G (Fig. 3F) suggested population bottlenecks or recent selective sweeps.

Genome-wide selection scans using π ratio and *F*_ST_ were performed between Clades E and G, excluding the hybrid Clade I to avoid confounding effects from its admixed background. We identified 2885 and 1018 candidate regions via π ratio (Fig. 3G) and *F*_ST_ (Fig. 3H), respectively, with 126 overlapping regions detected across all 18 chromosomes (Table S17). These overlapping regions yielded 78 candidate genes, 72 of which were functionally annotated (Table S18). This pervasive differentiation across the genome is consistent with the extreme pairwise *F*_ST_ (0.942) observed between the two lineages.

### Symbiosis-related genes’ expression profiling suggests divergent strigolactone signaling in *G. elata* clades

To investigate transcriptional variation among populations, transcriptome profiling was conducted. A total of 1615 genes were upregulated and 1899 genes were downregulated in Clade E compared to Clade G (Table S19). Although no KEGG pathways were significantly enriched, GO enrichment analysis identified 25 and 7 significantly enriched GO terms in Clades E and G, respectively (Fig. S10, Table S20). In Clade E, enriched GO terms included genes involved in diterpenoid biosynthesis (GO: 0016102), sesquiterpene biosynthesis (GO: 0051762), and terpene synthase activity (GO:0010333). Other enriched molecular functions included carbon–oxygen lyase activity (GO: 0016838), hydrolase activity (GO: 0016798 and GO: 0004553), and mannan endo-1,4-β-mannosidase activity (GO: 0016985) (Fig. S10A, Table S20). In contrast, Clade G exhibited enrichment in genes regulating cell shape (GO: 0008360), including glycosyltransferase (GO: 0016757), hexosyltransferase (GO: 0016758), and transmembrane receptor protein tyrosine kinase activities (GO: 0004714) (Fig. S10B, Table S21).

**Figure 4:**
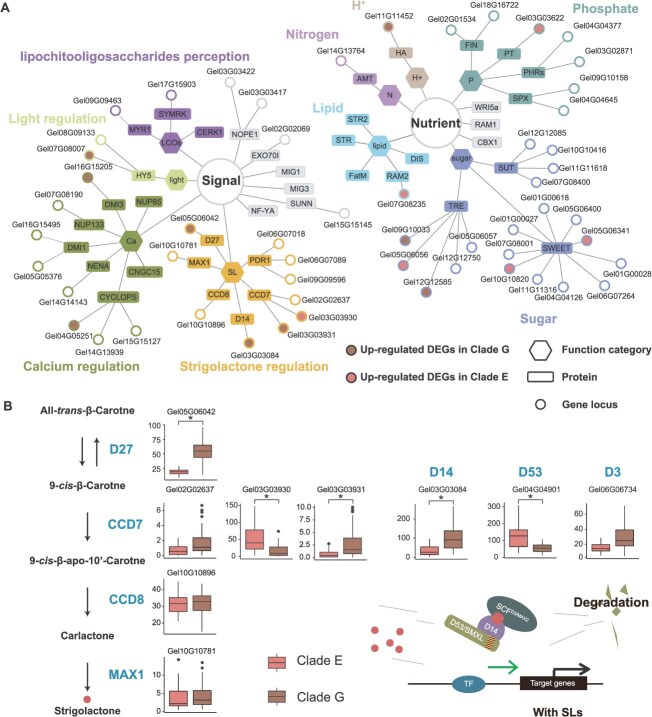
Expression of SRGs and divergence in SL signaling across *G. elata* clades. (A) Identification of SRGs and differential expression analysis. (B) Differential expression of genes involved in SL biosynthesis and perception pathways. *Y*-axis indicates FPKM values; ^*^P < 0.05.

AS *G. elata* forms orchid mycorrhizal (OM) associations with *Armillaria* fungi, expression differences in symbiosis-related genes (SRGs) were investigated. A total of 41 SRGs were compiled from previous studies (Table S22), and 55 SRG homologs were identified in the *G. elata* genome (Fig. 4A, Table S23). Most nutrient transport-related SRGs were present, except for those associated with lipid metabolism. Only *Required for Arbuscular Mycorrhization symbiosis 2* (*RAM2*), involved in converting acyl-CoA to sn-2 *monoacylglycerol (*β*MAG),* was retained. All genes in the common symbiosis signal pathway (CSSP) were identified (Fig. 4A). Among the SRGs, 15 were differentially expressed between Clades G and E. Notably, four out of ten SL pathway genes and three of five trehalases showed differential expression, suggesting divergence in SL and sugar signaling between clades.

SL biosynthesis begins with *DWARF27* (*D27*)-mediated isomerization of β-carotene to 9-*cis*-β-carotene, followed by sequential cleavage by *CAROTENOID CLEAVAGE DIOXYGENASE 7* (*CCD7*) and CAROTENOID CLEAVAGE DIOXYGENASE 8 (*CCD8*), and oxidation by *MORE AXILLARY GROWTH 1* (*MAX1*) to produce active SLs [26]. Of the two *CCD7* genes, *GeCCD7-2* (Gel03G03930) was highly expressed, while *GeD27* showed strong expression in Clade G (Fig. 4B). In SL signaling, the D14-D53 module regulates downstream transcription. D53 acts as a repressor until SL-bound D14 triggers D3-dependent degradation [26]. *GeD53* exhibited significantly higher expression in Clade E, whereas *GeD14* showed higher expression in Clade G. No significant differences were observed in *GeD3* expression (Fig. 4B).

### Clade-specific D14-D53 haplotypes underlie structural and functional divergence in strigolactone signaling

**Figure 5:**
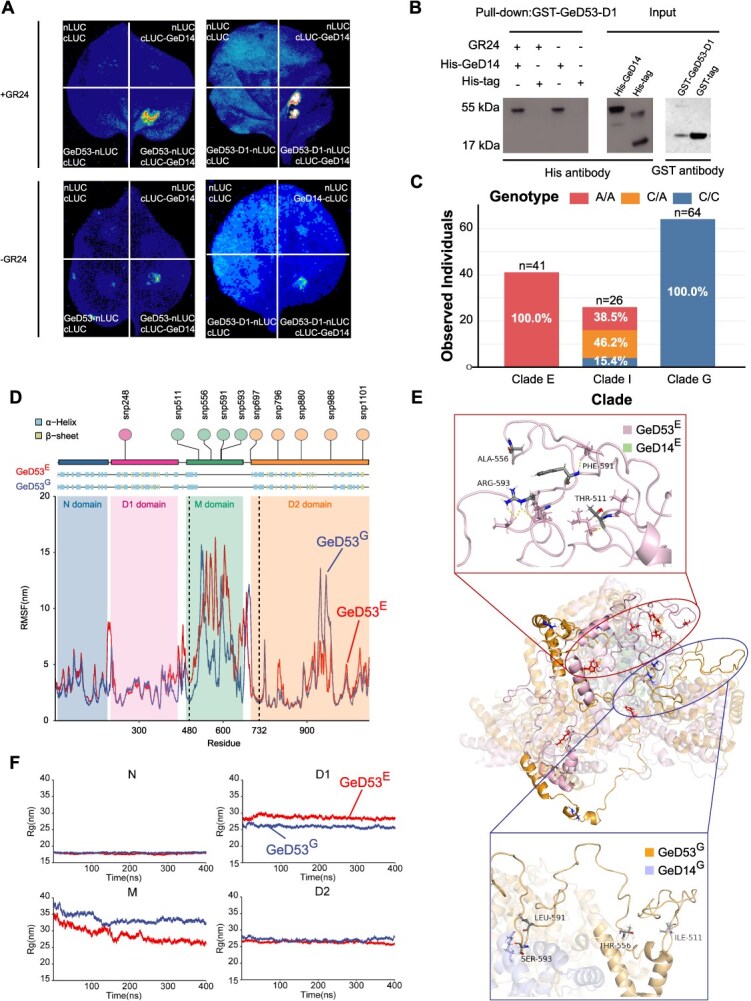
Haplotype-dependent functional divergence of D14-D53 complex modulates SL signaling efficiency. (A) LCI assay between GeD14^G^ and GeD53^G^. (B) Pull-down assay between GeD14^G^ and the GeD53^G^-D1 domain. (C) Proportions of D14 haplotypes across *G. elata* clades. (D) RMSF of the two GeD53 haplotypes. (E) Structural models of the GeD14-GeD53 complexes for the two haplotypes. (F) Rg of two GeD53 haplotypes.

Given the clade-specific expression patterns of *GeD14* and *GeD53*, their physical interaction was first confirmed using luciferase complementation imaging (LCI) assays. Both genes were cloned from the *G. elata* f. *glauca* genome assembly material (Fig. 5A). The GeD53 protein comprises four functional domains: an N-terminal domain, D1 ATPase domain, M domain, and D2 ATPase domain. Previous studies in rice and *A. thaliana* have shown that the D1 domain is critical for GR24-dependent D53-D14 binding [27]. Consistent with this, LCI and pull-down assays confirmed that the GeD53 D1 domain mediates its interaction with GeD14 (Fig. 5A and B).

The divergent expression of *GeD14* and *GeD53*, along with pronounced population differentiation, suggests that these genes segregate as distinct haplotypes across *G. elata* clades. At *GeD14*, a non-synonymous SNP was identified that resulted in a leucine-to-isoleucine substitution at position 88 (Table S24). This polymorphism exhibited fixed differences between Clade E and Clade G, while Clade I exhibited segregation with 46.2% heterozygous individuals (Fig. 5C). At *GeD53,* ten nonsynonymous mutations were identified, all in complete linkage disequilibrium (LD) with the *GeD14* variant (Table S24). No non-synonymous mutations were detected in *GeD3*. Based on these SNPs, *G. elata* samples were grouped into two distinct haplotypes: Hap E (*GeD14^Ile88^* and *GeD53* variants, hereafter *GeD14^E^* and *GeD53^E^*) and Hap G (*GeD14^Leu88^* and *GeD53* variants, hereafter *GeD14^G^* and *GeD53^G^*).

To evaluate the potential functional consequences of these genetic variations, two aspects were examined: (i) the impact of the Leu88Ile substitution on SL perception and (ii) haplotype-dependent variation in protein–protein interaction efficiency affecting SL signaling transduction. Hydrolase activity assays demonstrated that the substitution had minimal effect on SL perception. Yoshimulactone Green (YLG) hydrolysis revealed comparable catalytic activity between GeD14^G^ (*V*_max_ = 1.17, *K_m_* = 12.23) and GeD14^E^ (*V*_max_ = 0.3, *K_m_* = 9.95) (Fig. S11). Molecular dynamics (MD) simulations showed that the key catalytic residues His, Ser, and Asp are conserved between *GeD14* and *AtD14* and that the Leu88Ile mutation is located far from the active site (Fig. S11). Binding free energies of GeD14 proteins with GR24 were −28.18 kcal/mol (GeD53^E^-GR24) and −24.72 kcal/mol (GeD53^G^-GR24), suggesting no significant difference in SL binding affinity.

However, binding free energy calculations from the final 100 ns of MD simulations revealed differences in protein–protein interaction stability. The binding free energy of the GeD53^E^-GeD14^E^ complex was −187.74 kcal/mol, whereas that of GeD53^G^-GeD14^G^ was significantly lower at −285.54 kcal/mol, indicating greater binding stability and potentially more efficient SL signal transduction in the GeD53^G^-GeD14^G^ complex.

Root-mean-square fluctuation (RMSF) analysis indicated the highest variability in the 480–732 residual region, corresponding to the M domain of GeD53 (Fig. 5D). This region also showed the most pronounced difference in fluctuation between the two haplotypes, highlighting its importance in GeD53-GeD14 interaction (Fig. 5D). Structural alignment further showed that this region in GeD53^F^ adopts a more relaxed and open conformation compared to GeD53^E^ (Fig. 5E). The increased flexibility may be attributed to hydrogen bond formation between residues ARG-593, PHE-591, THR-511, and others in GeD53^E^, which were absent in GeD53G. Additionally, GeD53^E^ exhibited two extra α-helices in the D1 domain. Radius of gyration (Rg) analysis showed no significant differences in the D2 and N domains between haplotypes, but the D1 domain exhibited greater Rg variation, supporting structural divergence between the haplotypes (Fig. 5F).

### Haplotype-structured phenotypic divergence and co-expression networks of *GeD14*-*GeD53* in *G. elata*

**Figure 6:**
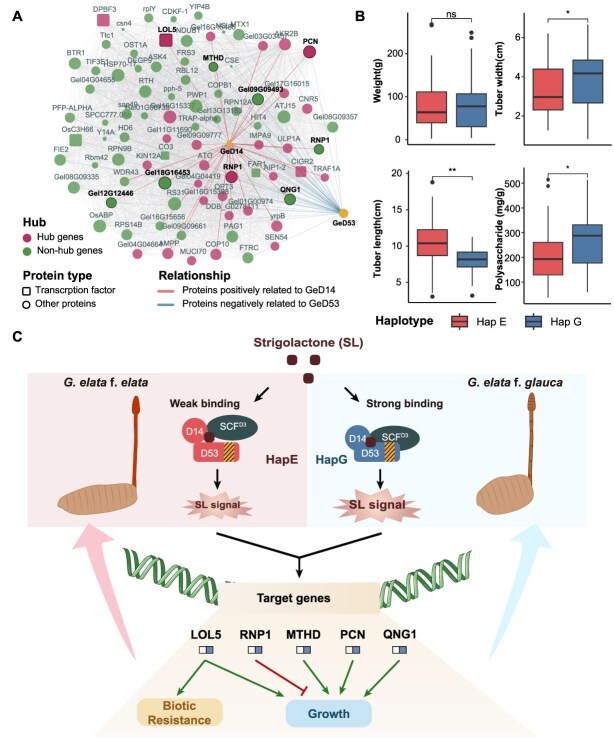
Haplotype divergence in the *GeD14-GeD53* network mediates growth and biotic resistance in *G. elata.* (A) Co-expression network of the darkturquoise module identified by WGCNA. Gene names in boldface represent those positively correlated with D14 and negatively correlated with D53. (B) Divergence in tuber morphology metrics and polysaccharide content between haplotypes. ^*^P < .05; ^**^P < .01. (C) Proposed model illustrating how haplotype-specific regulation via GeD14-GeD53 and its downstream target genes balances vegetative growth and stress resistance in the *G. elata–Armillaria* symbiosis. Colored squares below gene names indicate the clade of highest expression (red: Clade E; blue: Clade G).

To elucidate downstream regulatory networks of the *GeD14-GeD53* module, a weighted gene co-expression network analysis (WGCNA) was conducted. Among the 49 identified co-expression modules, *GeD14* and *GeD53* were assigned to the darkturquoise and darkmagenta modules, respectively (Fig. S12). Notably, 64.8% (59/91) of genes negatively correlated with *GeD53* expression were also present in the darkturquoise module, suggesting antagonistic regulation between *GeD14* and *GeD53*. This module contained 87 genes, including nine that displayed dual correlations—positively correlated with *GeD14* and negatively corelated with *GeD53* (Fig. 6A, Table S25).

Functional characterization of the darkturquoise module revealed that these co-regulated genes represent key regulators of the growth–defense trade-off. Three hub genes were identified within the module. The transcription factor *LOL5*, an ortholog of rice *OsLOL2*, is known to enhance pathogen resistance while suppressing plant growth when overexpressed [28], resembling the phenotypes observed in *d14* mutants [29]. *RNP1* (heterogeneous nuclear ribonucleoprotein 1) encodes a stress-responsive ribonucleoprotein that inhibits growth under abiotic stress conditions [30], and *MTHD* (3-hydroxyacyl-acyl carrier protein dehydratase) is essential for hypocotyl elongation, with silencing resulting in dwarf phenotypes [31]. In addition, *PCN* (*an auxin-responsive gene*) [32] and *QNG1* (*a queuosine salvage protein*) [33] were identified as key regulatory genes within the network. All five genes exhibited significantly higher expression levels in Clade G compared to Clade E (Table S26). The 2000-bp promoter regions of these hub genes were screened for the conserved D53-binding motif (5′-ATAACAA-3′). Multiple binding sites were identified in the promoters of MTHD (at 12 and −1681 bp), PCN (at 743 and 1292 bp), and QNG1 (at 928 bp). These results, coupled with their clade-specific expression patterns, suggest that GeD53 modulates tuber development by directly repressing these downstream effectors.

Phenotypic analyses further supported haplotype-structured divergence. Hap G developed shorter but wider tubers and accumulated higher levels of polysaccharides than Hap E individuals, despite having comparable fresh weights (Fig. 6B). These morphological and metabolic traits correlated with the haplotype-specific expression of *PCN*, *LOL5*, and *MTHD*, indicating coordinated regulation of tuber development.

Together, these findings demonstrate that the *GeD14-GeD53* module functions as a central regulator of growth–defense allocation in *G. elata* (Fig. 6C).

## Discussion

### The new chromosome-level genome of *G. elata* enriches the understanding of intraspecies variation of mycoheterotrophic plants

MHPs are ecologically and physiologically unique, and recent advances in sequencing technologies have enabled deeper comparative genomic analyses [9, 11, 34]. The independent evolution of mycoheterotrophy in at least 40 lineages [35] underscores the complexity in tracing its evolutionary origins. In this study, it was estimated that the fully mycoheterotrophic *Gastrodia* genus and the partially mycoheterotrophic *Dendrobium* genus diverged ~54.11 Mya (Fig. 1B). This divergence history differs from a prior study on partially mycoheterotrophic *Platanthera zijinensis* and fully mycoheterotrophic *Platanthera guangdongensis*, which estimated a much later divergence around 11.63 Mya [11], further suggesting considerable variation in evolutionary timelines among MHPs. Despite this distinct evolutionary trajectory, *K_s_* distribution analysis indicated that *G. elata* shares a conserved ancestral polyploidy event with other orchids, underscoring the importance of investigating genomic divergence at a finer scale to resolve how complex heterotrophic traits are maintained or lost within a single species.

While much research has focused on the differences between heterotrophic and autotrophic plants, intraspecies genomic variation within MHPs remains underexplored. Due to its genetic richness, *G. elata* provides a valuable model for such investigations. In this study, three *de novo* assemblies were generated to assess intraspecies diversity. Although the assemblies shared a substantial degree of synteny, ~25% of syntenic regions were inverted, and orphan genes were primarily located within these inversion regions. The significant enrichment of TEs at inversion breakpoints, coupled with the overrepresentation of orphan genes in these regions, suggests that transposon-mediated rearrangements serve as a primary driver of genomic divergence in *G. elata*. Such inversions may create ‘evolutionary islands’ by suppressing local recombination, thereby facilitating the accumulation of lineage-specific orphan genes or contributing to gene loss [36, 37]. These SVs potentially underpin the ecological specialization and phenotypic diversity observed among different cultivars.

### Genomic and transcriptomic divergence in cultivated *G. elata* populations

Population genomic analyses revealed substantial genetic differentiation between the two primary cultivated forms of *G. elata*, designated Clade E and Clade G, supporting the recognition of *G. elata* f. *glauca* and *G. elata* f. *elata* as distinct genetic clades. The intermediate Clade I exhibited elevated heterozygosity and π values, suggesting it may represent a product of human-mediated hybridization, an interpretation supported by its admixed ancestry in ADMIXTURE analysis. The low genetic diversity observed in Clades G and E (π < 0.1, heterozygosity <7%) is consistent with *G. elata*’s self-pollinating reproductive strategy and likely domestication bottlenecks, further supported by negative Tajima’s *D* values. These findings provide genomic evidence for the taxonomic distinction between the ‘Brown Tianma’ (Clade G) and ‘Red Tianma’ (Clade E), with implications for germplasm conservation and breeding.

Transcriptomic analysis also identified clade-specific expression profiles. In particular, terpene synthase genes (particularly monoterpene synthase 8; Tables S13 and S19) were more highly expressed in Clade E. These genes are involved in the biosynthesis of volatile sesquiterpenes and monoterpenes [38], which have been shown to inhibit the growth of *Armillaria spp.* in *G. elata* [39].

### *GeD53* structural variation fine-tunes strigolactone signaling through D14-D53 interaction dynamics

Furthermore, investigation into SRGs revealed that the SL signaling components *GeD14* and *GeD53* exhibited clade-specific expression patterns. Structural analyses identified distinct haplotypes for each gene. D14, an α/β-fold hydrolase, is a central regulator of plant–fungi symbiosis and is essential for the perception of AM fungi and subsequent infection [40]. Kinetic analyses indicated that the two GeD14 haplotypes displayed similar *K_m_* values, suggesting conserved ligand-binding affinity. Although the Leu88Ile substitution in *GeD14* had minimal impact on SL perception—consistent with its location distal from the active site residues Ser104, His254, and Asp226 [41]—the interaction dynamics between *GeD14* and *GeD53* were functionally significant.

D53 is the primary downstream effector of D14 [27, 42–44]. MD simulations revealed that the GeD14-GeD53 complex in Clade G exhibited greater structural stability, which was associated with increased GeD53 degradation and stronger SL signaling. These findings align with the observed upregulation of *GeD14* and downregulation of *GeD53* in Hap G (Fig. 4B), indicating elevated SL signaling activation in this clade.

To further analyze the structural basis for these interaction differences, the domain architecture of GeD53 was analyzed. This protein comprises four domains: the N-terminal region, D1 ATPase, M (bridge) domain, and D2 ATPase (Fig. 5C). Although the D1 ATPase domain is the primary functional domain [27], MD simulations suggested that haplotype-specific conformational differences were largely confined to the M domain, a loop-rich region known to influence protein flexibility and ligand-binding dynamics (Fig. 5D) [45]. The M domain of GeD53^E^ formed more intramolecular interactions than GeD53^G^. For instance, residue 511 in GeD53^E^ is a polar threonine (T), capable of forming hydrogen bonds, while the corresponding position in GeD53^G^ is occupied by a non-polar isoleucine (I). This substitution likely results in a more compact structure for GeD53^E^, weakening its binding affinity for GeD14^E^. These structural insights are supported by yeast two-hybrid (Y2H) assays, which showed that the D1 domain alone was sufficient for activity, whereas the D1M construct lacked interaction capacity [27], underscoring the regulatory influence of the M domain in *D14-D53* binding.

While transgenic validation is the standard approach, its application in *G. elata* is currently limited by the species’ unique mycoheterotrophic physiology and the lack of comparable tuber-forming contexts in autotrophic model plants. As an alternative, we evaluated phenotypic variations across 150 accessions based on their haplotypes (Fig. 6B), consistent with emerging research on complex horticultural crops [46, 47]. These results provide preliminary evidence for the functional impact of these variants. While these population-level associations offer a meaningful framework for understanding SL signaling in *G. elata*, we look forward to future breakthroughs in genetic transformation that will allow for more direct functional characterization.

### Strigolactone-directed gene networks balance growth and biotic resistance in the *G. elata*-*Armillaria* symbiosis

The role of D53 as a transcriptional repressor in the SL signaling pathway [42–44], along with its D14-dependent degradation via the ubiquitin–proteasome system [44], suggests that genes positively correlated with *GeD14* but negatively correlated with *GeD53* are likely direct targets of SL-mediated transcriptional activation. Through WGCNA and differential expression analyses, *LOL5*, *RNP1*, and *MTHD* were identified as core regulatory hubs within the *GeD14*-associated co-expression module, indicating their potential involvement in SL-dependent coordination of growth and symbiosis.

Notably, different *G. elata* cultivars have been found to be associated with distinct *Armillaria* lineages across Chinese regions [48], implying clade-specific symbiotic adaptations. It is proposed that haplotype divergence in the *GeD14*-G*eD53* module may contribute to such ecological specialization by fine-tuning transcriptional networks. This hypothesis is further supported by the presence of conserved D53-binding motifs (5′-ATAACAA-3′) in the promoters of *MTHD*, *PCN*, and *QNG1*, which exhibit clade-specific expression patterns correlating with the contrasting tuber phenotypes observed (Fig. 6B). Specifically, Wang *et al.* [43] and Ye *et al.* [49] have confirmed that D53 directly targets downstream genes via this core motif to regulate biological processes; thus, the enhanced stability of the GeD14^G^-GeD53^G^ interaction likely facilitates more efficient recruitment of the GeD53 suppressor to these target promoters. The morphological and metabolic differences—such as variations in tuber size and polysaccharide accumulation—likely reflect distinct resource allocation strategies, modulated by *GeD14-GeD53* interactions, to optimize either vegetative growth or stress tolerance in response to environmental contexts, thereby representing a key adaptive mechanism for the evolution and maintenance of obligate mycoheterotrophy. To integrate these findings, a working model is proposed (Fig. 6C) wherein haplotype-specific divergence in SL-mediated transcriptional networks governs a trade-off between growth and biotic resistance during mycoheterotrophic symbiosis.

However, whether the observed differences in tuber morphology and carbohydrate accumulation directly influence symbiotic efficiency remains to be determined. Future studies should quantify mycorrhizal colonization rates and fungal-derived nutrient transfer across different haplotypes to clarify the functional consequences of SL-regulated developmental and metabolic traits. Additional mechanistic insights into how these traits mediate *Armillaria* interaction would further clarify how obligate mycoheterotrophs maintain a balance between developmental and pathogen resistance.

## Materials and methods

### Plant materials

A *G. elata* Bl. f. *glauca* individual used for *de novo* genome assembly was collected from Xiaocaoba in Yunnan Province, China. For population genomics, 150 individuals were sampled from four distinct geographical regions: Guizhou (*n* = 57), Hubei (*n* = 27), Shaanxi (*n* = 25), and Yunnan (*n* = 41), with representative coordinates provided in Table S27.

Mature tuber samples were collected from planting holes, which are specialized fungal cultivation beds layered with hardwood sticks and *Armillaria* fungus. To ensure genetic representativeness and avoid clonal sampling, only one individual was collected per hole, with a minimum distance of 5 m maintained between adjacent holes.

### DNA library construction and sequencing

The scape of the *G. elata* plant was used for genome sequencing. Genomic DNA was extracted using the modified CTAB method [50], and its quality and quantity were assessed with a Qubit fluorometer (Thermo Fisher Scientific). Short-insert libraries (350 bp) were prepared using the NEB Next® Ultra DNA Library Prep Kit for Illumina® (NEB, USA) following the manufacturer’s instructions and sequenced in 150-bp paired-end mode on an Illumina NovaSeq 6000 platform.

For long-read sequencing, genomic DNA was fragmented to 20 Kb and used to construct a SMRT bell CLR library according to the manufacturer’s instructions (Pacific Biosciences, CA, USA). Sequencing was performed on the PacBio Sequel II platform. Additionally, a Hi-C library was generated using crosslinked chromatin digested with *Dpn* II and sequenced on the NovaSeq 6000.

### Genome assembly and quality assessment

*De novo* assembly of PacBio SMRT reads was performed using FALCON (v0.3.0) [51], and scaffolds were polished with Illumina paired-end reads using Pilon (v.1.23) [52]. High-throughput chromosome conformation capture (Hi-C) reads were aligned to the polished assembly using BWA (v0.7.17) [53], and scaffolds were anchored to chromosomes using the 3D-DNA pipeline (v180419) [54], with further manual refinement in Juicebox (v1.11.08) [55]. Chromosome nomenclature followed previously reported *G. elata* assemblies based on syntenic relationships [16].

### Genome annotation

Repeat elements were annotated using the extensive *de novo* TE annotator (EDTA) (v2.0.0) [56] pipeline under sensitive mode. This pipeline uses RepeatModeler (v2.0.2a) [57] for the *de novo* identification of TEs. The genome was subsequently masked using RepeatMasker (v4.1.4) [58].

Protein-coding genes were predicted using a combined strategy incorporating *de novo* prediction, homology-based annotation, and transcript evidence. (i) RNA-seq data (Table S28) were aligned to the unmasked genome using HISAT2 (v2.1.0) [59] and assembled into transcripts with StringTie (v2.1.4) [60]. Additionally, TRINITY (v2.8.5) [61] was employed for the *de novo* transcriptomic assembly. PASA2 (v2.5.2) [62] was then employed to generate the transcript evidence. (ii) For *de novo* prediction, BRAKER2 pipeline [63] was used with GeneMark-ET (v4.33) [64] and AUGUSTUS (v3.4.0), incorporating RNA-seq alignments. (iii) For homology-based annotation, GenBlastA (v1.0.1) [65] and GeneWise2 (v2.4.1) [66] were used to align protein-coding genes from related species to the unmasked *G. elata* genome. GeMoMa (v1.8) [67] was also employed to infer gene models based on previously published *G. elata* annotations. (iv) Gene models from all approaches were integrated using EVidenceModeler (v1.1.1) [68]. To validate completeness, miniprot [69] was used to confirm that genes annotated in prior *G. elata* assemblies [10, 14, 16] were also captured in this annotation.

SRGs were identified by querying known SRG sequences (Table S17) against the *G. elata* proteome using BLASP (*E*-value < 1 × 10^−5^, identity >40%, subject coverage >40%). Candidate proteins were further verified using HMMER to confirm the presence of relevant Pfam domains using default parameters.

### Comparative genomics analysis

To identify SVs, three *G. elata* assemblies were aligned using minimap2 (-ax asm5), and syntenic relationships were assessed via SyRI (v1.70) [70]. Syntenic regions were defined by SYN tags, while rearrangements were classified as inversions (INV, INVTR, INVDP) or translocations (TRANS). TE density in 10-kb windows flanking each breakpoint was compared with a null distribution generated from 1000 randomly sampled genomic regions using the Wilcoxon rank-sum test. Additionally, inversions larger than 1 Mb were manually validated via Hi-C contact maps to exclude assembly artifacts (Fig. S6).

Protein-coding genes from 15 plant species (Table S29) were clustered into gene families using OrthoMCL [71] (parameters: percentMatchCutoff = 50, evalueExponentCutoff = 5). Shared orthologous groups among the three *G. elata* assemblies were identified using OrthoMCL. Single-copy orthologs present in all 15 species were used to construct a phylogenetic tree. Protein-coding nucleotide sequences were aligned based on their protein translations using MACSE [72]. Aligned sequences from each gene family were concatenated to infer a molecular clock using MCMCtree [73]. Time calibration points were obtained from TimeTree (http://www.timetree.org/) and included the following: *Amborella trichopoda vs*. other plants (173–199 Mya), monocotyledons *vs*. dicotyledons (148–173 Mya), *Vitis vinifera vs*. *Arabidopsis thaliana* (105–115 Mya), *Oryza sativa vs*. *Ananas comosus* (94–117 Mya), *A. officinalis vs*. Orchidaceae (93–118 Mya), and the subfamily *Vanilloideae vs*. other *Orchidaceae* subfamilies (77–84 Mya).

To investigate the WGD history, protein-coding sequences from *G. elata*, *D. chrysotoxum*, *A. officinalis*, and *A. calamus* were used for all-to-all alignments using BLASTP with an E-value cutoff of 1 × 10^−5^. Based on the identified homologous gene pairs, syntenic blocks within each genome were identified using WGDI [74]. Prior to *K_s_* calculation, tandem duplicate genes were filtered out using the Duplicate_gene_classifier module implemented in MCScanX to eliminate potential false-positive WGD signals. For each syntenic block, the *K_s_* of each gene pair was calculated using the PAML package. The median *K_s_* value of all gene pairs within a syntenic block was utilized to represent the *K_s_* of that block. The frequency distribution of *K_s_* values across all blocks was then calculated, and Gaussian mixture modeling was applied to fit the *K_s_* peaks based on the distribution characteristics.

Gene family expansion and contraction were assessed using CAFE5 [75], with significance defined as *P* < 0.05. Expanded families were subjected to KEGG enrichment analysis using the ClusterProfiler package [76]. For contracted families, the longest protein in each family was selected and annotated via BLASTP against the Swiss-Prot database.

### Resequencing and SNP calling

Genomic DNA was extracted from mature tubers of 150 *G. elata* individuals using the modified CTAB method [50] and sequenced on the Illumina NovaSeq 6000 platform as previously described to an average depth of 5× per sample. Raw sequencing reads were processed and filtered using fastp [77] to obtain clean reads. These were aligned to the *G. elata* genome using BWA-MEM [53]. Duplicate reads were removed, and BAM files were sorted before variant calling. GATK HaplotypeCaller [78] was employed to generate individual gVCF files, which were then merged using GATK GenotypeGVCFs. A hard filter was applied to the merged VCF file with the following parameters: QD ≥ 2.0 && FS ≤ 60.0 && MQ ≥ 40.0 && MQRankSum ≥ −12.5 && ReadPosRankSum ≥ −8.0). SNPs located within 5 bp of InDels, with a missing rate > 0.7, or a minor allele frequency (MAF) < 0.05, were excluded from further analysis.

### Population genetic analysis

Kinship analysis was conducted using PLINK (v2.00) [79] on the imputed VCF file with the --make-king-table option. Kinship coefficients were interpreted as follows: values <0 indicated no familial relationship; 0.0442–0.0884 indicated a relationship within three generations; 0.0884–0.177, within two generations; 0.177–0.354, full siblings; and >0.354, clonal individuals [80]. Samples with a kinship coefficient >0.177 were excluded from subsequent analyses.

LD was analyzed using PopLDdecay [81], with a maximum distance of 300 kb. SNP pruning was performed by selecting the *r^2^* at which LD decayed to 50% of its maximum. Pairwise genetic distances were calculated based on pruned SNPs using VCF2Dis [82], with 1000 bootstrap replications. A neighbor-joining (NJ) tree was constructed from the distance matrix using PHYLIPNEW. *Gastrodia menghaiensis* was used as the outgroup, and its sequencing data were obtained from the SRA database (accession: SRX11022665).

The pruned SNP dataset was also used for PCA conducted with PLINK (v1.90) [83] and for population structure analysis using ADMIXTURE [84], with 10-fold cross-validation and 10 bootstrap replicates.

Based on population structure results, π and pairwise genetic differentiation (*F*_ST_) were calculated using pixy [85] in non-overlapping 10-kb windows. Tajima’s D was calculated with vcftools [86], and sample heterozygosity was assessed using PLINK (v1.90).

### Transcriptome analysis

Total RNA was extracted from mature tubers of the same individuals using the QIAGEN RNeasy Plant Mini Kit (QIAGEN, Hilden, Germany). RNA-seq libraries were prepared using the TruSeq RNA Library Preparation Kit (Illumina, CA, USA), and 150-bp paired-end sequencing was performed on the Illumina Novaseq 6000 platform. After filtering with fastp [77], reads were aligned to the *G. elata* genome using STAR [87]. Gene-level quantification was performed using htseq-count v2.0.4 [88].

To ensure statistical reliability, samples were grouped according to their identified clades and haplotypes (Table S16). Differential expression analysis was subsequently conducted between specific groups, including comparisons between Clade G (*n* = 71) and Clade E (*n* = 49), as well as between Hap G (*n* = 64) and Hap E (*n* = 41), using DESeq2 [89]. Differentially expressed genes (DEGs) were identified based on the thresholds of FDR < 0.05 and |log_2_(fold change)| > 1. Enrichment analysis was conducted using the clusterProfiler R Package [76].

A WGCNA was conducted through the WGCNA R Package. Hub genes were identified via the ‘chooseTopHubInEachModule’ function. To identify specific downstream effectors, a threshold based on the WGCNA adjacency matrix (weight > 0.7) was applied within the target modules.

### Yoshimulactone Green (YLG) hydrolysis assays

The full-length cDNAs of *AtD14*, *GeD14^E^,* and *GeD14^F^* were cloned into the pET32a vector to generate TRX-His_6_ fusion proteins. These were expressed in Rosetta-gami 2(DE3) cells (Coolaber, China) and induced with 0.2 mM isopropyl β-D-thiogalactoside (IPTG) at 16°C for 16 hours. Fusion proteins were purified using Ni-NTA Resin (Sangon, China), following the manufacturer’s protocols, and quantified using the Bio-Rad protein assay reagent.

*In vitro* YLG hydrolysis assays were performed in 96-well black plates with 200 μl reaction mixtures containing 1× PBS buffer (pH 7.3), 15 μg AtD14, GeD14^E^, or GeD14^G^, and YLG at final concentrations of 2, 4, 8, 10, 12, 16, 18, 20, 40, and 60 μM. After incubation in the dark at 26°C for 3 h, fluorescence intensity was measured at an excitation wavelength of 480 nm and an emission wavelength of 520 nm. 

The Michaelis constant (*K_m_*) was determined using the following formula:


$$ {K}_m=\frac{V_{\mathrm{max}}\left[S\right]}{V}-\left[S\right], $$

where ${K}_m$ represents the enzyme’s affinity for the substrate, ${V}_{\mathrm{max}}$ is the maximum fluorescence intensity, $\left[S\right]$ is the substrate concentration, and $V$ is the observed fluorescence at that concentration.

### Pull-down assays

The cDNA encoding the D1 domain of *GeD53* was cloned into the pGEX4T-1 vector. The expression and purification of GST and GST-GeD53 followed the same procedure as for GeD14, described above. Fusion proteins were purified using GST-tag Resin (Sangon, China) following the manufacturer’s protocols and quantified using the Bio-Rad protein assay reagent. The expression and purification of GeD14 were carried out as described previously.

After purification, a pull-down assay was performed by incubating 50 μl of GST resin, 10 μg of purified GeD14 protein, 20 μg of purified GeD53-D1 protein, and GR24 (when applicable) in 300 μl binding buffer (10 mM Tris–HCl, 0.1 M NaCl, 1 mM DTT, pH 7.6) with gentle shaking at 4°C for 1 hour. The resin was then washed five times with binding buffer containing 0.5% Tween-20. After washing, 50 μl of SDS-PAGE sample buffer was added, and the mixture was denatured at 95°C for 5 minutes. The samples were subjected to 10% SDS-PAGE gel, and proteins were detected using an HRP-conjugated Anti-6×His Tag mouse monoclonal antibody (Sangon, China) and visualized with an enhanced chemiluminescence reagent (Beyotime, China).

### LCI assays

LCI assays were performed using the pCAMBIA-nLUC and pCAMBIA-cLUC vectors [90] to assess protein–protein interactions in *Nicotiana benthamiana* leaves. The full-length GeD53 and its D1 domain (GeD53-D1) were fused to the nLUC vector, while GeD14 was fused to the cLUC vector.

Constructed vectors were introduced into *Agrobacterium*. Prior to infiltration, the bacterial cultures were resuspended in an infiltration buffer (0.2 mM acetosyringone, 10 mM MES) to an optical density of OD_600_ = 0.5. Equal volumes of *Agrobacterium* strains carrying nLUC and cLUC constructs were mixed and co-infiltrated into the leaves of *N. benthamiana* using a needleless syringe. Plants were incubated at 24°C for 48 hours after infiltration.

Luciferase signals were visualized using a low-light cooled CCD imaging camera (Princeton Instruments). Prior to imaging, leaves were sprayed with 1 mM luciferin and incubated in the dark for 15 minutes. The experiment was repeated for three to eight independent biological replicates.

### Molecular dynamics simulation

Initial protein structures of the GeD14-GeD53 complexes were generated using AlphaFold3 [91]. All simulations were conducted using AMBER 22 [92]. The AMBER ff19SB force field was applied to the proteins, and the mbondi2 radii were utilized for the system. Each complex was solvated in an OPC water model and neutralized with Na^+^ ions.

The Sander module was employed for energy minimization, heating, and equilibration steps. Each system underwent extensive energy minimization, followed by gradual heating from 0 to 300 K. Pre-equilibration was performed under the isothermal-isobaric (NPT) ensemble. Formal production simulations were carried out for 500 ns for each system. To ensure statistical robustness, five independent replicate simulations (500 ns × 5) were performed for each haplotype complex.

Trajectory analyses were performed to evaluate conformational dynamics, including RMSF to identify high-variability regions (e.g. the M domain) and *R*_g_ to assess structural compactness. The final 100 ns of the production trajectories were used to calculate the binding free energy (Δ*G*_bind_) using the MM/GBSA method to evaluate the stability of the GeD14-GeD53 interaction. Hydrogen bonding interactions and structural alignments were analyzed to identify key residues driving the divergence between haplotypes. All visualizations were generated using PyMOL (version 3.0).

## Supplementary Material

Web_Material_uhag099

## Data Availability

The raw genomic sequencing data and transcriptome sequencing data are available in the National Genomics Data Center (NGDC) under PRJCA037379. The genome assembly and gene annotation reported in this paper have been deposited in the Genome Warehouse in the National Genomics Data Center, Beijing Institute of Genomics (China National Center for Bioinformation), Chinese Academy of Sciences, under accession number GWHBHOU00000000, which are publicly accessible at https://ngdc.cncb.ac.cn/gwh/. Additional supporting datasets, including population SNP vcf files, are available at Zenodo (https://doi.org/10.5281/zenodo.18217218).
