# Supplementary material for: Chromosome-level genome assembly and population genomics unveil strigolactone-regulated growth adaptation in the mycoheterotrophic orchid Gastrodia elata
Source: Hortic Res. 2026 Jun 4;13(7):uhag099. doi: 10.1093/hr/uhag099 (PMC13282667; doi:10.1093/hr/uhag099)
Supplement: Web_Material_uhag099 [file web_material_uhag099.zip › supplementary_figures.docx]

## Supplementary Figures


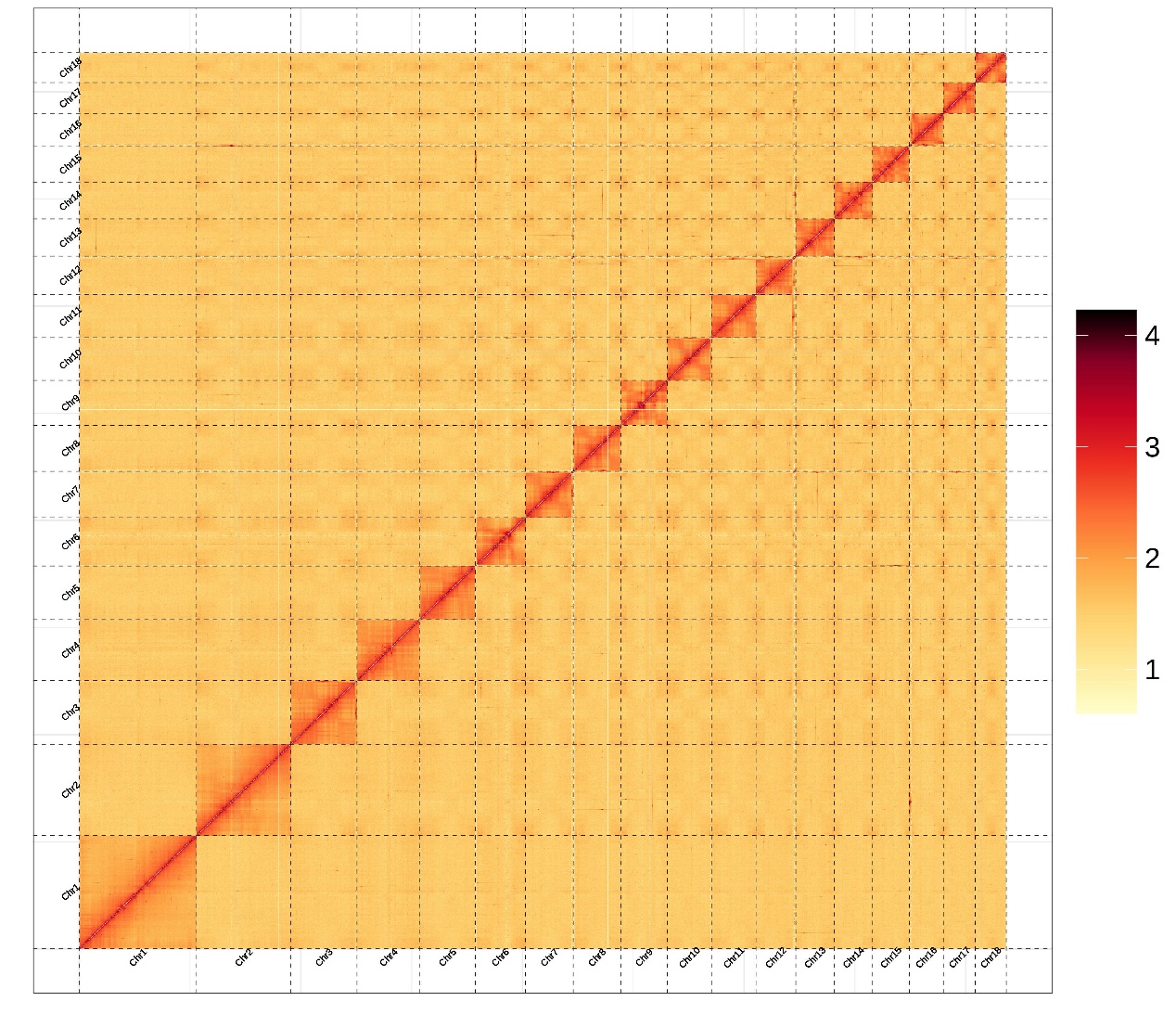


**Figure S1** Hi-C heat map of chromosome interactions.


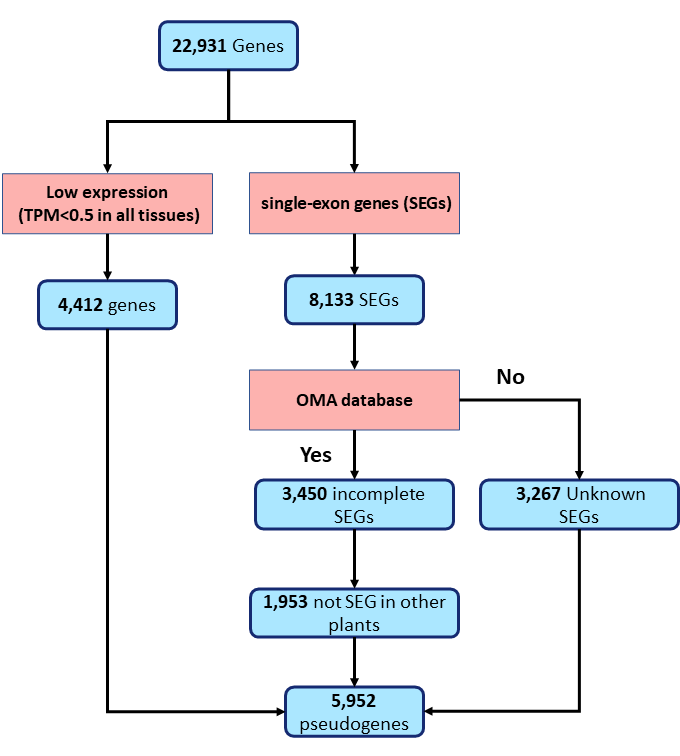


**Figure S2** The pseudogene filtering process for the genome of *G. elata*.


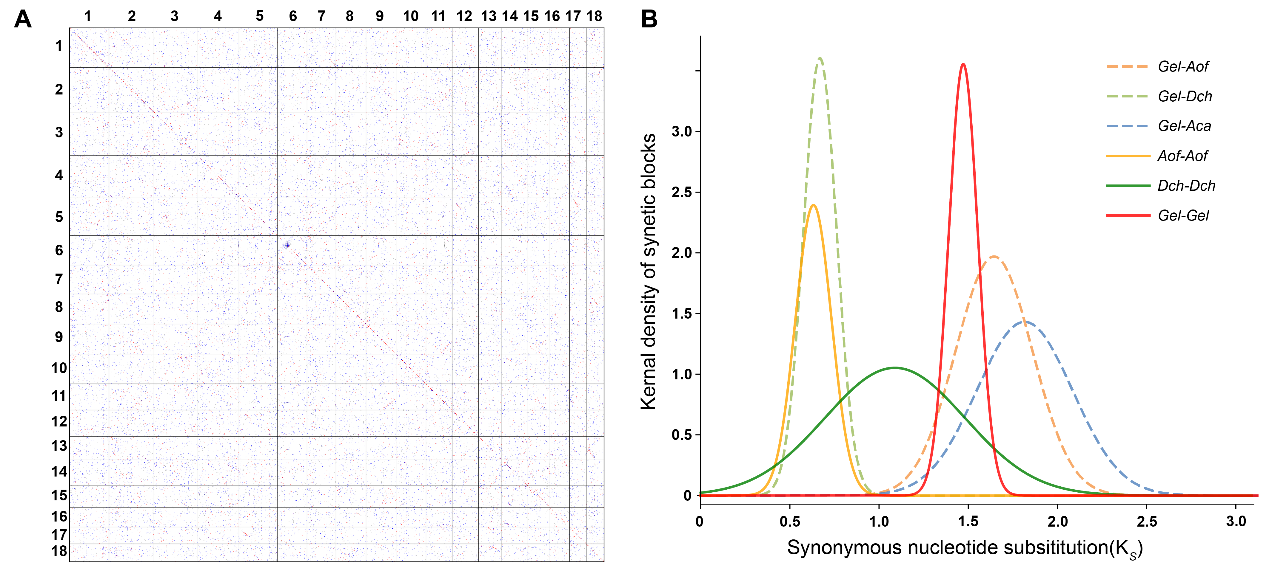


**Figure S3** Whole-genome duplication (WGD) analysis of *G. elata*.**(A)** Distribution of paralogous gene pairs within the *G. elata* genome. **(B)** Kernel density distributions of synonymous substitution rates (*K*s) for syntenic gene pairs. Solid lines represent intragenomic (paralogous) comparisons within *G. elata* (*Gel*–*Gel*, red), *Asparagus officinalis* (*Aof*–*Aof*, orange), and *Dendrobium chrysotoxum* (*Dch*–*Dch*, green). Dashed lines represent intergenomic (orthologous) comparisons between *G. elata* and *A. officinalis* (Gel–Aof, orange), *D. chrysotoxum* (Gel–Dch, olive), and *Acorus calamus* (Gel–Aca, blue).


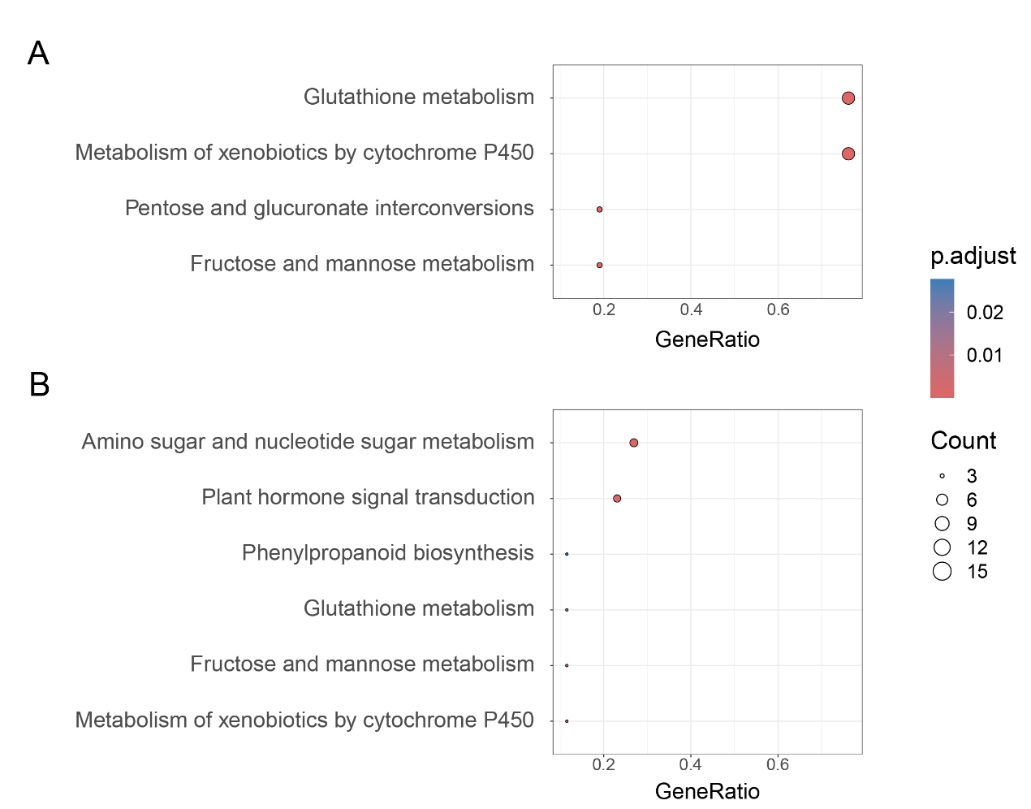


**Figure S4** The KEGG enrichment of expanded gene families. **(A)** Enrichment results for expansion genes in the genus *Gastrodia*. **(B)** Enrichment results for expansion genes in the genus *Gastrodia elata*.


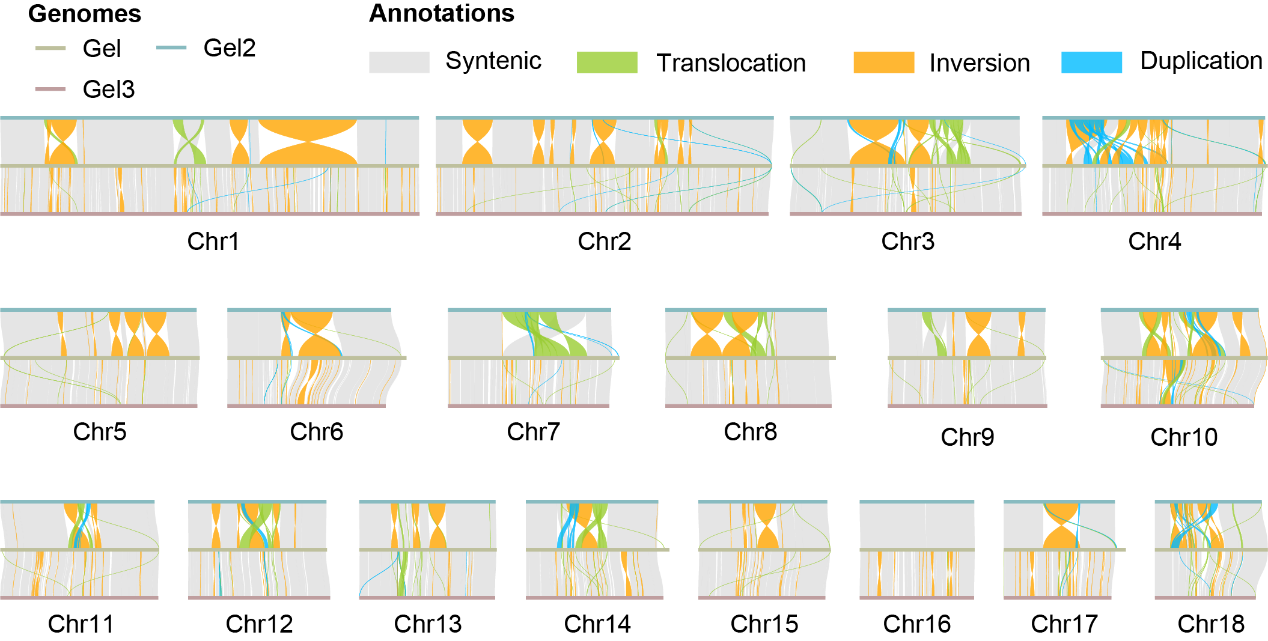


**Figure S5** The structural variations among *G. elata* genome assemblies.


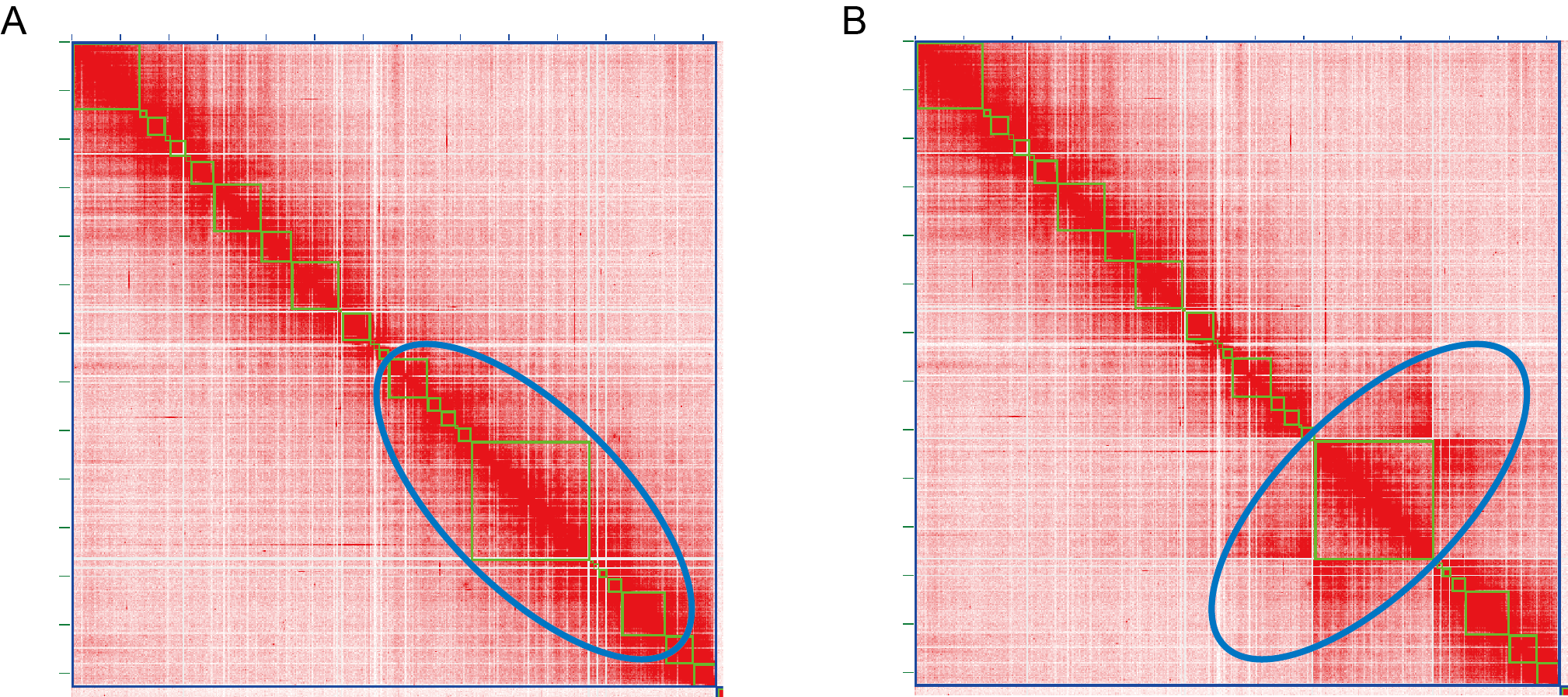


**Figure S6** The Hi-C heatmap illustrating the inversion in the 83 Mb-113 Mb region on chromosome 1 **(A)** assembly results from this study. **(B)** direction adjusted according to the assembly reported by Xu et al**.** The corresponding inversion region are highlighted with blue circles.


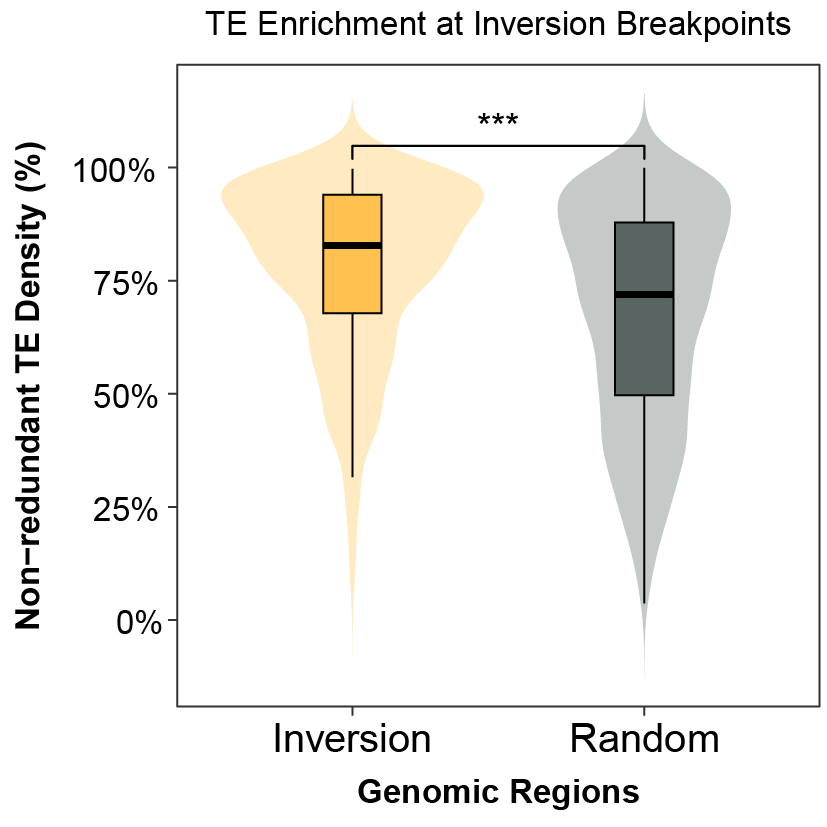


**Figure S7** Enrichment of transposable elements (TEs) at inversion breakpoints. Comparison of non-redundant TE density within 10-kb flanking regions of inversion breakpoints “Inversion”) versus 1,000 randomly sampled genomic windows (“Random”). ****p* < 0.001


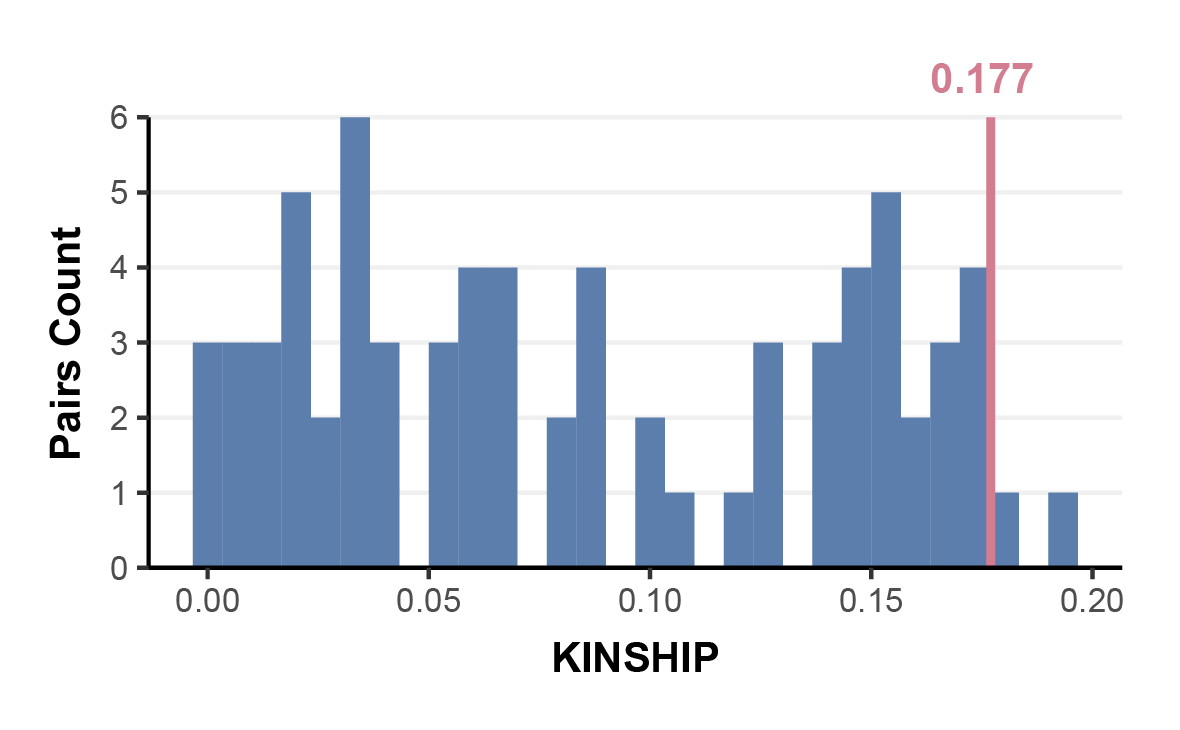


**Figure S8** Distribution of kinship coefficients greater than zero in the *G. elata* population.


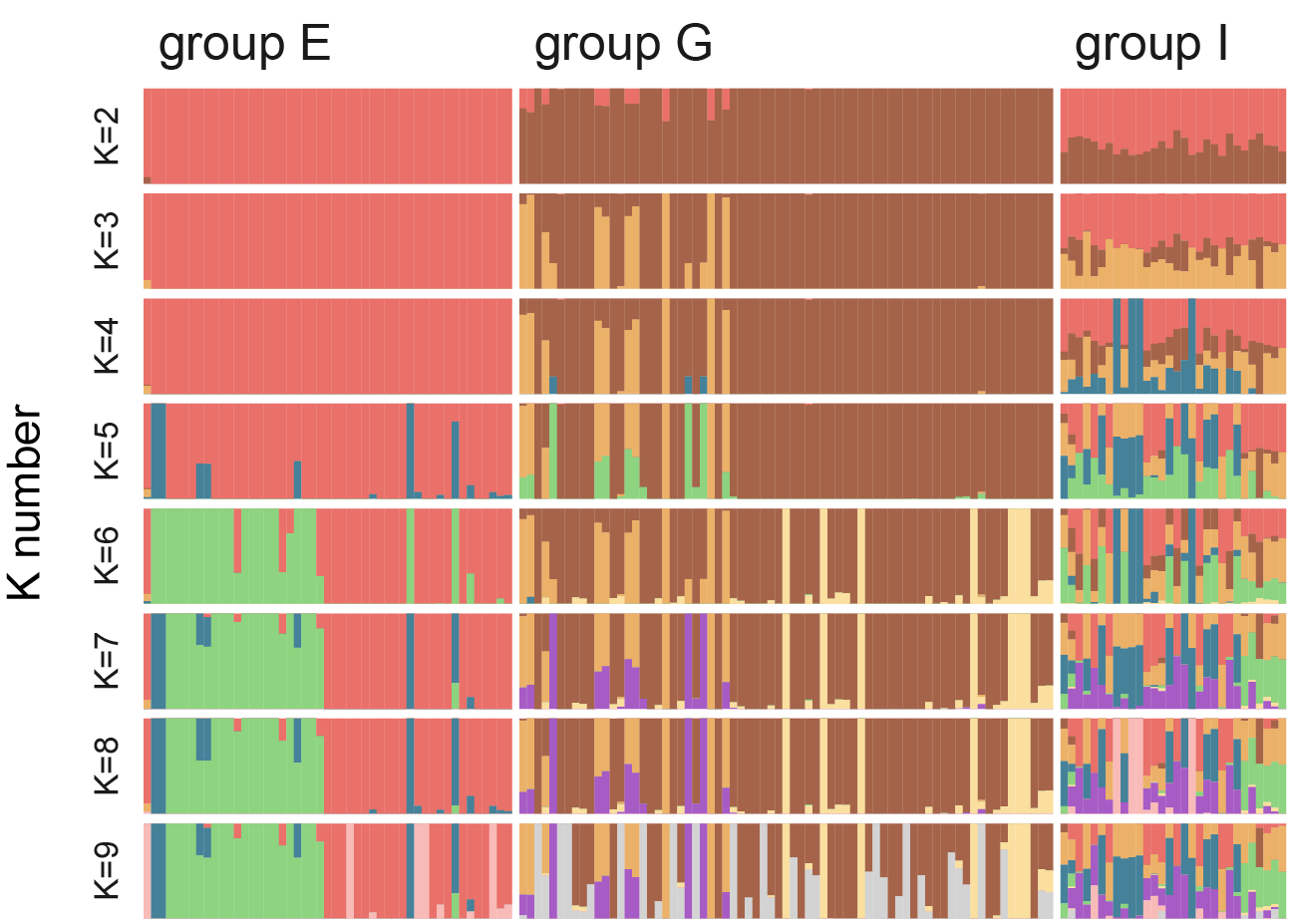


**Figure S9** ADMIXTURE analysis for K from 2 to 9.


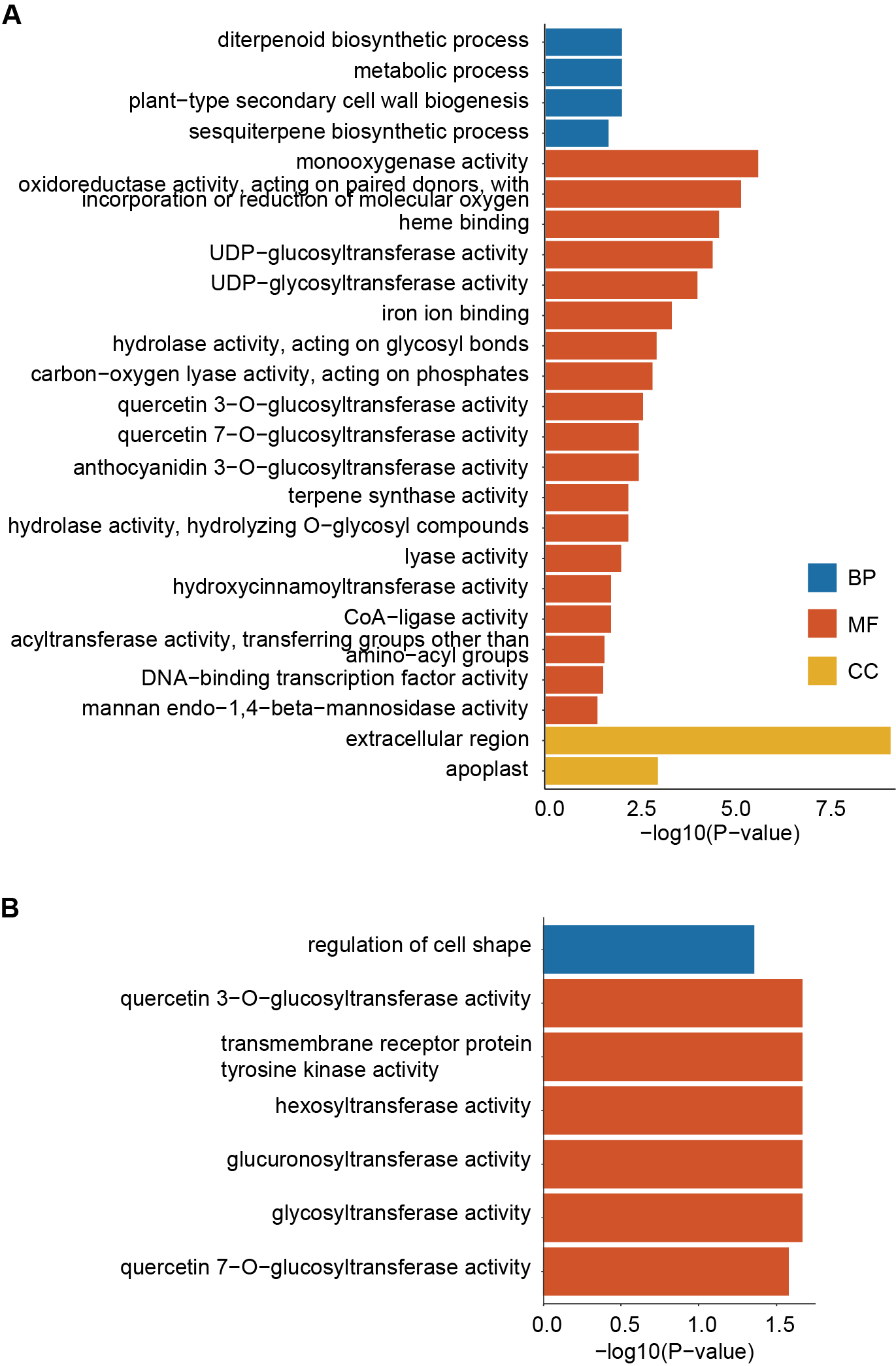


**Figure S10** GO enrichment analysis for differently expressed genes between Clade G and Clade E. **(A)** up-regulated gene in Clade E. **(B)** up-regulated gene in Clade G.


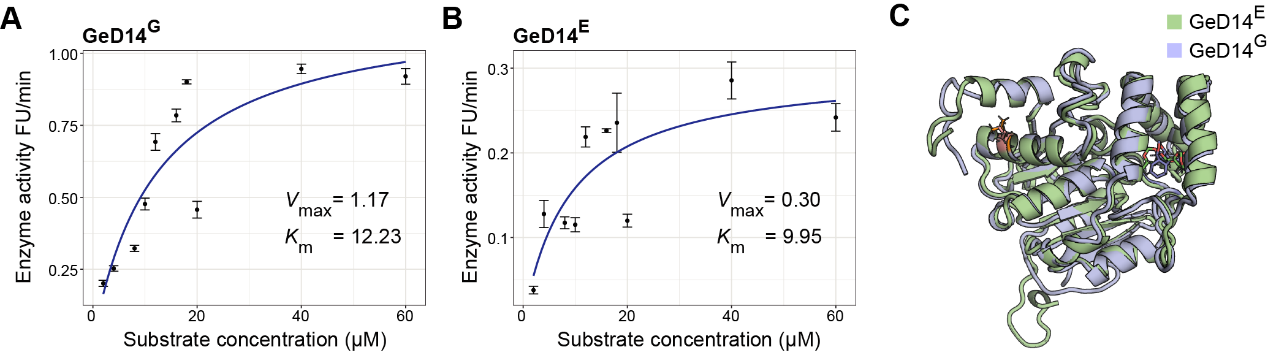


**Figure S11** Hydrolytic activity of **(A)** GeD14^G^ and **(B)** GeD14^E^ on YLG Substrate. **(C)** The representative conformation of GeD14^G^-GR24 and GeD14^E^-GR24 complex.


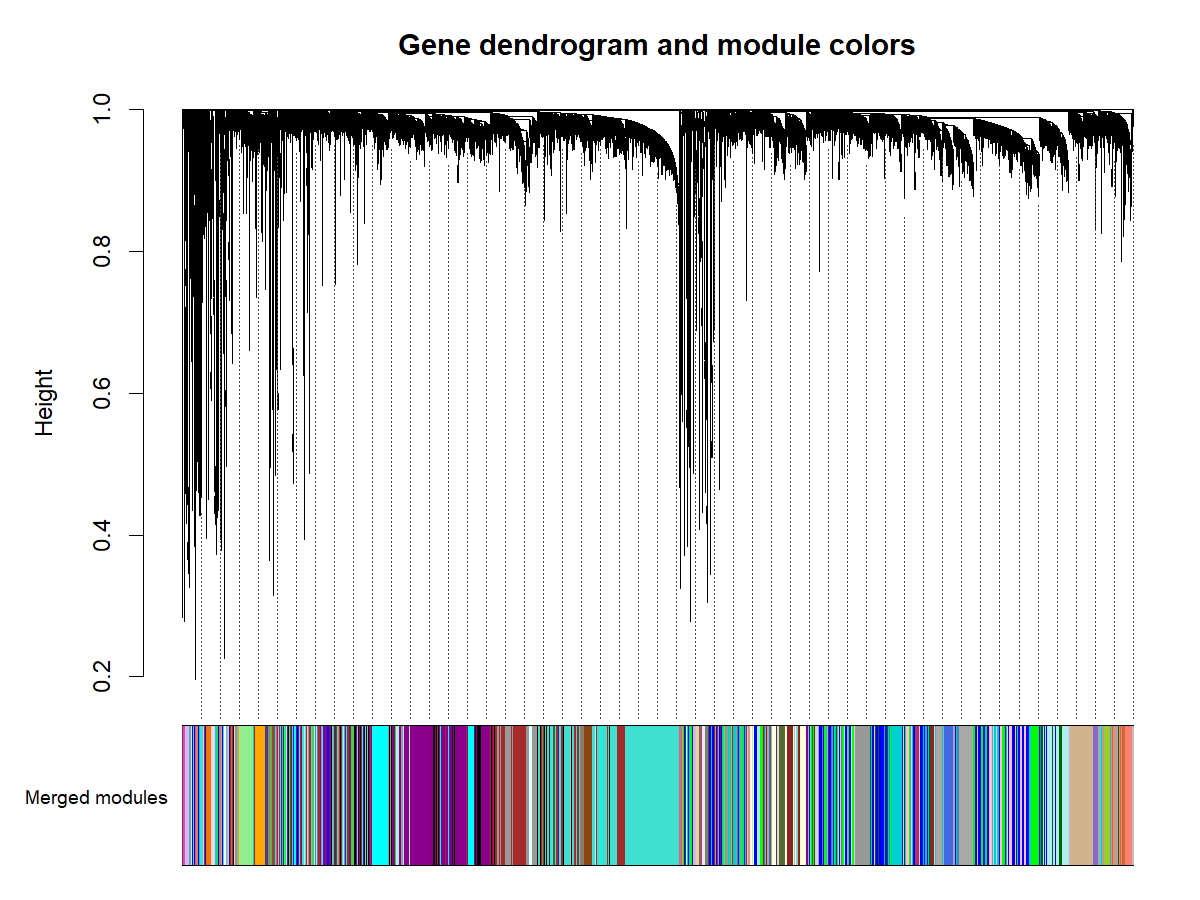


**Figure S12** WGCNA module identification
